# Determination of novel reference genes for improving gene expression data normalization in selected canine reproductive tissues – a multistudy analysis

**DOI:** 10.1186/s12917-020-02635-6

**Published:** 2020-11-12

**Authors:** Marta Nowak, Selim Aslan, Mariusz P. Kowalewski

**Affiliations:** 1 Institute of Veterinary Anatomy, Vetsuisse Faculty, University of Zurich (UZH), Winterthurerstrasse 260, CH-8057 Zurich, Switzerland; 2 Department of Obstetrics and Gynecology, Faculty of Veterinary Medicine, Near East University, Nicosia, North Cyprus Turkey

**Keywords:** Reference genes, Dog (*Canis lupus familiaris*), Reproductive tract

## Abstract

**Background:**

Real time RT-PCR (qPCR) is a useful and powerful tool for quantitative measurement of gene expression. The proper choice of internal standards such as reference genes is crucial for correct data evaluation. In female dogs, as in other species, the reproductive tract is continuously undergoing hormonal and cycle stage-dependent morphological changes, which are associated with altered gene expression. However, there have been few attempts published so far targeted to the dog aimed at determining optimal reference genes for the reproductive organs. Most of these approaches relied on genes previously described in other species. Large-scale transcriptome-based experiments are promising tools for defining potential candidate reference genes, but were never considered in this context in canine research.

**Results:**

Here, using available microarray and RNA-seq datasets derived from reproductive organs (corpus luteum, placenta, healthy and diseased uteri) of dogs, we have performed multistudy analysis to identify the most stably expressed genes for expression studies, in each tissue separately and collectively for different tissues. The stability of newly identified reference genes (*EIF4H*, *KDELR2*, *KDM4A* and *PTK2)* has been determined and ranked relative to previously used reference genes, i.e., *GAPDH, β-actin* and *cyclophillin A/PPIA*, using RefFinder and NormFinder algorithms. Finally, expression of selected target genes (luteal *IL-1b* and *MHCII*, placental *COX2* and *VEGFA*, and uterine *IGF2* and *LHR*) was re-evaluated and normalized. All proposed candidate reference genes were more stable, ranked higher and introduced less variation than previously used genes.

**Conclusions:**

Based on our analyses, we recommend applying *KDM4A* and *PTK2* for normalization of gene expression in the canine CL and placenta. The inclusion of a third reference gene, *EIF4H*, is suggested for healthy uteri. With this, the interpretation of qPCR data will be more reliable, allowing better understanding of canine reproductive physiology.

**Supplementary Information:**

The online version contains supplementary material available at 10.1186/s12917-020-02635-6.

## Background

Among the final steps of large-scale expression experiments, like microarray or RNA-seq (Next Generation Sequencing, NGS), are analysis and validation of data [1, 2]. Therefore, expression of chosen candidate genes of interest is investigated by real-time RT-PCR (qPCR) with higher numbers of biological replicates. While qPCR has become a routine and well-established method for gene expression analysis, data normalization still remains problematic and is subject to frequent criticism [3–5]. The most often applied normalization strategy is the use of internal controls, i.e., reference genes [6]. These genes are supposed to be stably expressed in the examined tissues and among experimental groups, and, if properly validated, allow controlling for errors that might be introduced during the procedure. Furthermore, it has to be emphasized that proper normalization is of utmost importance since it also allows overcoming of pitfalls related to sample preparation and processing. These include, i.a., RNA extraction technique, sample quality, applied DNase treatment and RT method. Thus, proper validation of reference genes is crucial for generating reliable data. Poor validation of reference genes can lead to overlooking of discrete changes in gene expression, thereby generating false data, or can result in misunderstanding of underlying biological processes [3].

For validation of transcriptome data derived from microarray or RNA-seq experiments, it is a good practice to use as reference genes the most stably expressed genes, according to the data set generated. Notably, transcriptome results provide information not only about differentially expressed genes (DEG), which are typically of primary research interest, but also about stably-expressed genes. The latter are often underrated and omitted by researchers, although they may include promising potential reference genes [7].

Regarding the canine species *(Canis lupus familiaris)*, available reference genes were adapted from other species [8–10]. Although these genes are not assumed to be “perfect” for normalization, they are constantly used for qPCR data evaluation, and include genes preferably selected from different functional families (e.g., glyceraldehyde-3-phosphate dehydrogenase (*GAPDH*), *β-actin*, *cyclophilin A* (*PPIA*), *18S rRNA*, or succinate dehydrogenase complex flavoprotein subunit A (*SDHA*)). Moreover, based on our literature search [8, 9], all previous attempts to evaluate potential reference genes in the dog, aimed at screening through various samples such as bone marrow, duodenum, heart, kidney, liver, lung or lymph nodes, were based on groups of animals that were heterologous with respect to breed, sex, age, body weight and health status. Finally, not surprisingly, the conclusion was drawn [8, 9, 11] that none of the evaluated genes would be universally suitable for normalization of gene expression in all canine tissues. These results led us to conclude that perhaps reliable reference genes should be validated first for particular organs or systems, such as the reproductive system which is our particular research interest.

Throughout the reproductive cycle, the female tract is continuously undergoing hormonal and morphological changes, associated with alternations of gene expression profiles that are tissue- and cycle stage-dependent. This is also characteristic of the domestic dog*,* which relative to other domestic animal species exhibits several species-specific features in its reproductive physiology (reviewed in [12–15]). Thus, dogs are monoestric, polytocous and aseasonal breeders. The reproductive cycle of a dog is comprised of four phases: proestrus, estrus, diestrus (i.e., the luteal phase) and an obligatory sexual inactivity phase, anestrus. Because the length of each phase is highly variable, the whole cycle can last from 5 up to 13 months [12, 13]. It presents unique hormonal and regulatory features reflected in differential gene expression among and within the particular reproductive organs. Due to this unique reproductive physiology, translational research is frequently limited. In more detail, proestrus is associated with strongly increasing estrogen (E2) levels secreted by growing follicles with a preovulatory peak as high as 120-140 pg/ml [12, 13, 16]. Towards the end of estrus, concentrations of E2 gradually decrease together with simultaneously increasing progesterone (P4) secreted from preovulatory luteinizing follicles [12, 15]. This triggers the final LH surge and leads to ovulation [12]. In the place of a ruptured follicle, corpora lutea (CLs) are formed and the luteal phase starts. It needs to be emphasized that the dog is the only domestic animal species that does not produce steroids in the placenta [17, 18]. In this context, CLs are the only providers of P4 in the dog, thus having a paramount role in the maintenance of canine pregnancy [15, 19]. Furthermore, dogs lack a luteolytic signal in the absence of pregnancy, resulting in a prolonged luteal phase in non-pregnant animals referred to as pseudopregnancy [15, 20]. Consequently, secretion of P4 in non-pregnant bitches lasts for a similar time as during pregnancy (around 60 days) or can even be prolonged beyond this time [13, 15]. In pregnant bitches luteal function is actively terminated shortly before parturition, at around day 60 of pregnancy [13, 15]. The lifespan of the CL is terminated by utero-placental prostaglandin F2α (PGF2α) in an acute process of prepartum luteolysis [17, 19, 21, 22]. The production of PGF2α and initiation of the prepartum luteolytic cascade are regulated at the level of the placental feto-maternal interface and are mediated by local, i.e., placental, availability of P4 [22, 23]. Interestingly, preterm luteolysis and, hence, abortion, can be induced by treatment with an antigestagen, e.g., aglepristone which blocks the P4 receptor (PGR), thereby initiating the placental luteolytic cascade [21, 22]. It has to be emphasized that within the canine placenta PGR are expressed only in its maternal part [22, 24], i.e., stroma-derived decidual cells, whereas the synthesis of PGF2α occurs in trophoblast, i.e., in the placenta fetalis [22]. A functional interplay between these two compartments of the placenta, the placenta materna and placenta fetalis, is thus responsible for initiation of the luteolytic cascade and thereby for maintenance of pregnancy, and initiation of parturition in the dog.

Altogether, there is a highly complex series of biochemical and morphological changes occurring in the reproductive tract throughout the cycle of the dog. Moreover, the distinctive physiological features of canine reproduction, such as prolonged steroidogenic activity of CLs and prolonged exposure of the endometrium to P4 in non-pregnant bitches, or lack of placental steroidogenesis, all indicate that application of reference genes from other species may not be suitable.

To our knowledge, there has been no previous attempt to perform multistudy analysis of available datasets from microarray and RNA-seq experiments derived from the canine species, in order to find novel, stably-expressed reference genes for normalization of gene expression studies in the canine reproductive organs. Therefore, here, by using data sets generated in our own laboratory and those from laboratories of other researchers, derived from luteal, placental and uterine (pregnant, non-pregnant, healthy and diseased) tissues, we attempted to search for, and validate, new reliable reference genes for future research.

## Results

### Tissue-specific candidates for reference genes

**Fig. 1:**
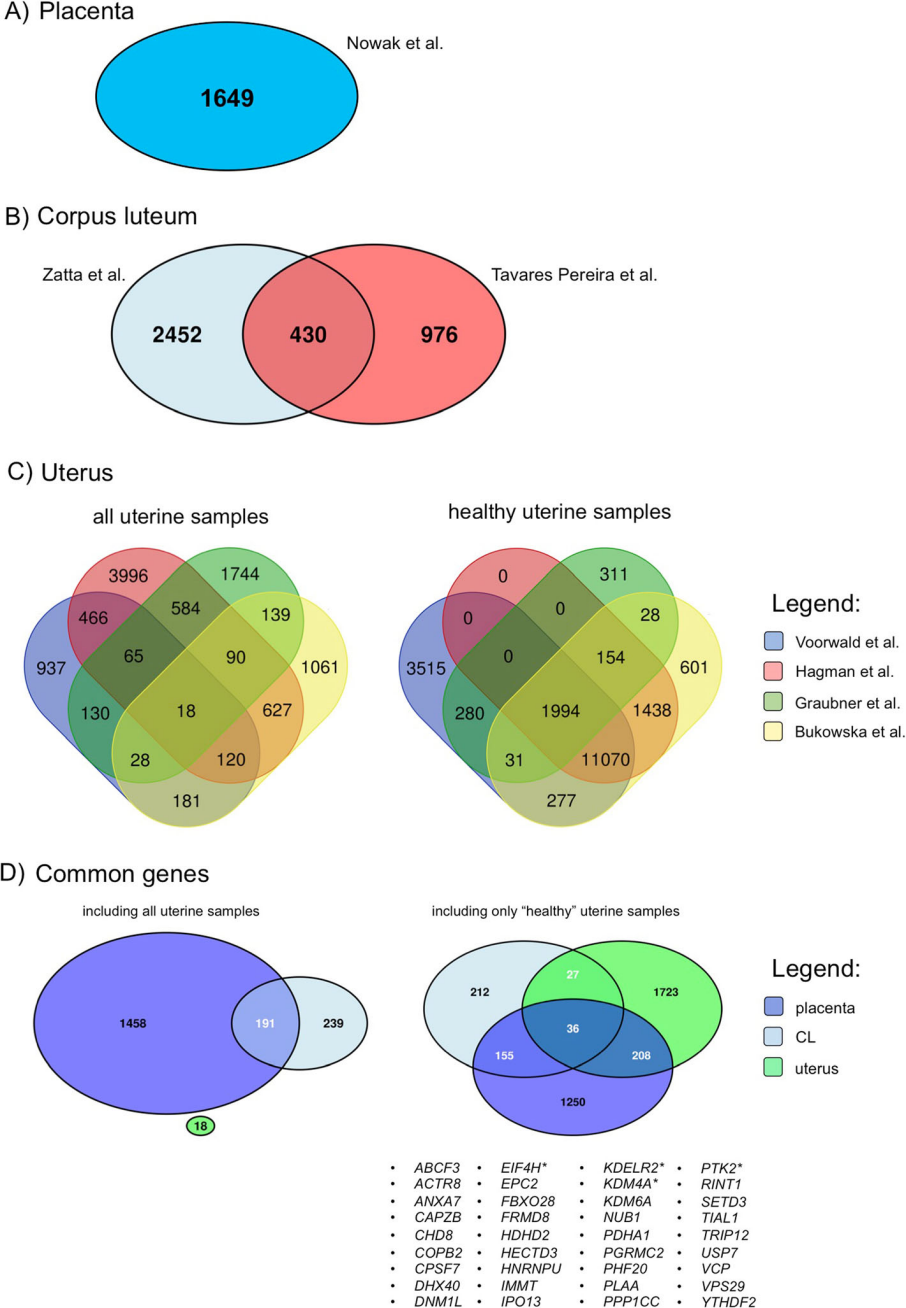
Stably-expressed genes in canine placenta (**a**), corpus luteum (**b**) and uterus (**c**) including different treatments, cycle/pregnancy stages and/or health and treatment status, filtered by the following criteria: the coefficient of variation (CV) < 0.2; the base mean value of number of transcripts > 500. **c** Analysis of uterine genes was performed with (i) all uterine samples and (ii) without diseased tissues (healthy uterine samples). **d** Common stable genes identified in data sets from all examined tissues. No gene was found when all uterine samples were included. However, when excluding pathological uterine samples, 36 potential candidates were identified common for all remaining samples. Asterisk (*) indicates genes chosen for further validation

Applying the criteria described in Methods, we identified 1649 potential candidates for reference genes for placenta (Fig. 1a), 430 for CL (Fig. 1b) and 18 for all uterine samples (Fig. 1c). The summary and lists of all identified genes in a particular dataset are also shown in Supplementary Material 2. When cumulatively compared, no gene was found to be common for all three tissue types examined under all conditions, i.e., with regard to treatments, cycle/pregnancy stage and health status (Fig. 1d). This made us conclude that high variations could be introduced by using uterine samples derived from animals with pathological conditions, i.e., pyometra, mucometra or CEH. Therefore, the analysis for uterus was repeated, excluding pathological samples, but keeping the controls from each uterine dataset. Subsequent analysis with only “healthy uterine samples” identified 1994 genes with potentially high stability in the uterus (Fig. 1c). Finally, these were compared with those genes identified in all placenta and CL samples (including antigestagen- and firocoxib- treated ones), and 36 genes were found to be common for all tissues (Fig. 1d). Out of these, four genes from different functional categories were selected for further validation and included: eukaryotic translation initiation factor 4H (*EIF4H*), endoplasmic reticulum lumen protein-retaining receptor 2 (*KDELR2*), lysine-specific demethylase 4A (*KDM4A*) and protein tyrosine kinase 2 (*PTK2*).

### Reference gene expression and stability

**Fig. 2:**
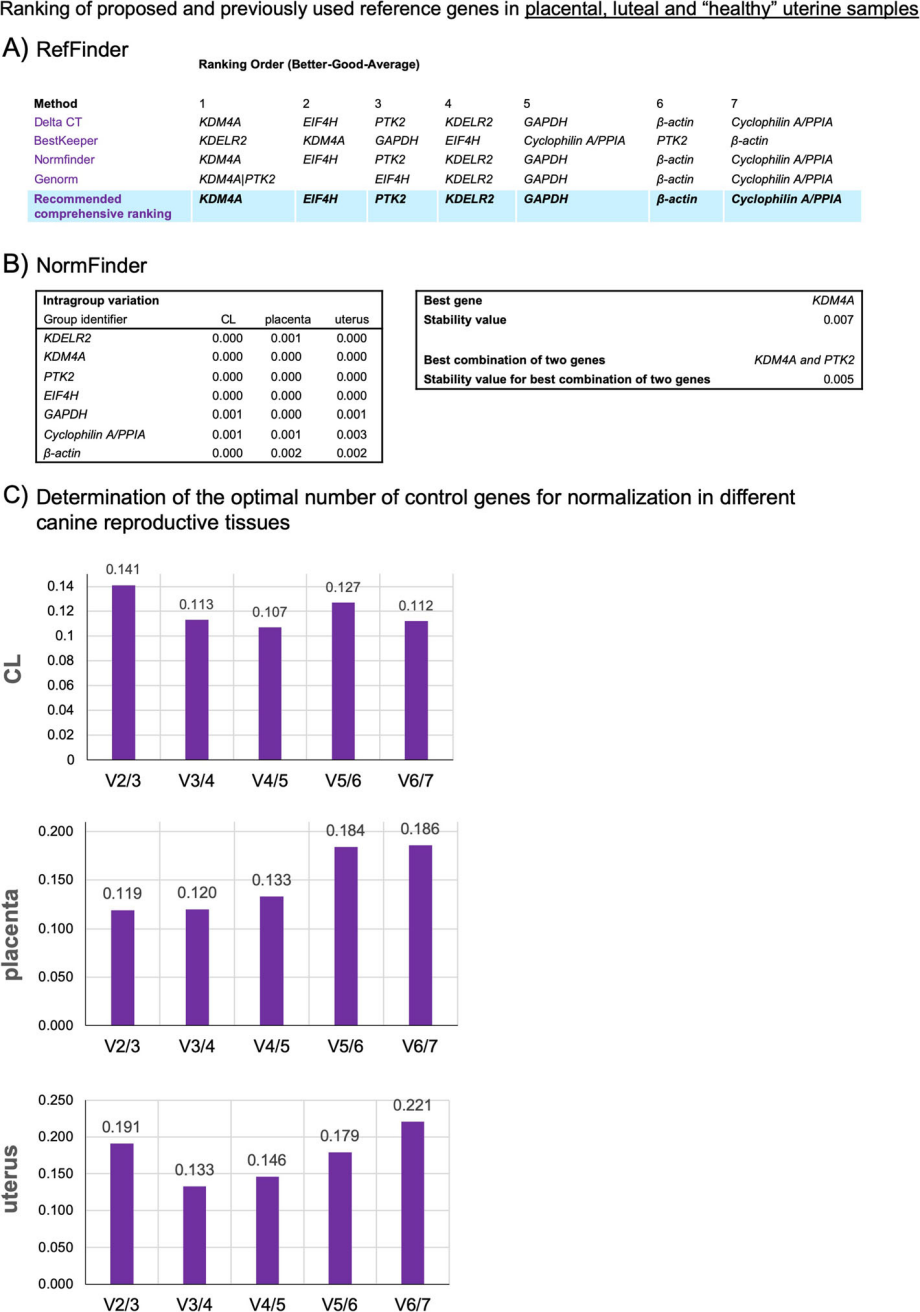
Ranking of proposed and commonly (previously) used reference genes in placental, luteal and healthy uterine samples, generated by RefFinder (**a**) and NormFinder (**b**) applications. The NormFinder tool was used to calculate intragroup (group refers to a particular tissue: 1-CL, 2-placenta, 3-uterus) stability values. Both softwares ranked *KDM4A*, *EIF4H* and *PTK2* as the most stably expressed genes. The comparison was made including previously used reference genes (*GAPDH*, *β-actin*, *cyclophilin A/PPIA*) (**c**) Determination of the optimal number of control genes for normalization in canine CL, placenta and uterus. Pairwise variation (V_n/n + 1_) analysis was done to determine the number of control genes required for accurate normalization. A cut-off value 0.15 was applied (Vandesompele et al. 2002). Pairwise variation analysis shows that V2/3 values in canine CL and placenta, and V3/4 in the uterus, were lower than 0.15, indicating that two reference genes are suitable for gene normalization in CL and placenta, but 3 genes should be included for uterus

Expression of the four (4) selected candidate genes as well as three (3) previously used reference genes was assessed by TaqMan RT-qPCR in 55 canine samples (uterine, luteal and placental; *details in Methods*). Next, this group of 7 genes was ranked according to their stability by RefFinder (Fig. 2a). *KDM4A*, *EIF4H* and *PTK2* were placed in comprehensive ranking as the most stable genes (Fig. 2a). Further, NormFinder recognized lower intragroup variations, i.e., those within a particular tissue (group identifier in Fig. 2b), when compared to previously used genes, and found KDM4A to be the most stable gene (Fig. 2b). PTK2 together with KDM4A was also identified as the best combination of genes by this software (Fig. 2b). Finally, pairwise variation analysis with GeNorm showed that, when following the recommended criteria for V_n/n + 1_ of 0.15 as the cut-off [25], two genes appear to be enough for normalization of gene expression data in CL and placenta, whereas 3 genes are sufficient for the uterus (Fig. 2c).

### Comparison of representative target gene expression normalized with previously used and newly determined reference genes

**Fig. 3:**
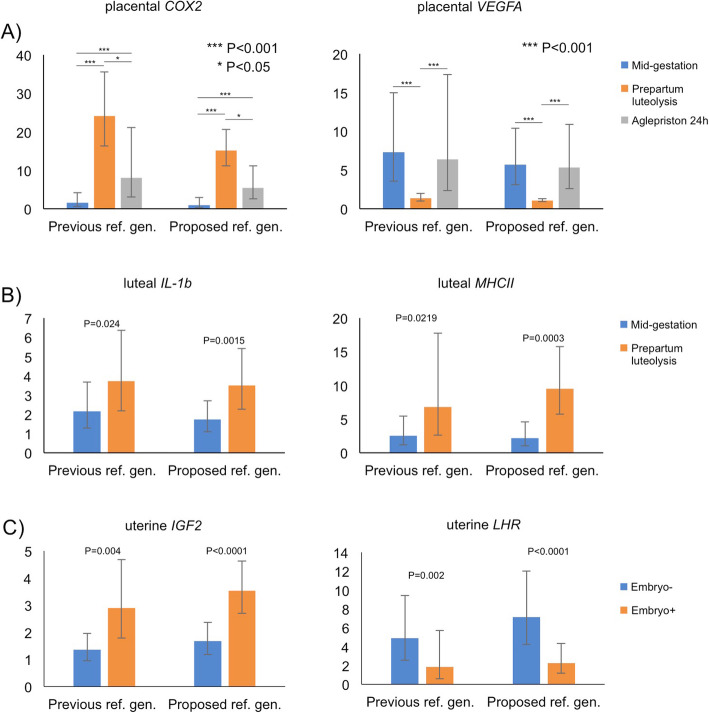
Validation of reference genes in the expression of exemplary target genes known to vary greatly in target tissues: (**a**) Placental expression of cyclooxygenase-2 (*COX2/PTGS2*) and vascular endothelial growth factor A (*VEGFA*) during mid-gestation, antigestagen-induced and normal luteolysis; (**b**) Luteal expression of interleukin 1b (*IL-1b*) and major histocompatibility complex class II molecules (*MHCII*) during mid-gestation and normal prepartum luteolysis; (**c**) uterine expression of insulin-like growth factor 2 (*IGF2*) and luteinizing hormone receptor (*LHR*) in non-pregnant uterus (Embryo-) and during early pregnancy (Embryo+). All experiments were normalized with commonly (previously) used reference genes (Previous ref. gen.: *GAPDH*, *β-actin*, *cyclophilin A/PPIA*) or proposed reference genes (Proposed ref. gen.: *KDM4A*, *EIF4H* and *PTK2*), ranked as the best normalizers by RefFinder and NormFinder applications, as determined by real-time (TaqMan) qPCR. Data are presented as Xg ± geometric standard deviation (SD). Normalization with routinely used genes resulted in higher intragroup variation when compared to results normalized with newly proposed genes

Expression of several genes known to vary from previous studies was evaluated and normalized using either three previous reference genes or the three best reference genes indicated from the current analysis (i.e., KDM4A, EIF4H and PTK2). Following the V_n/n + 1_ analysis, the KDM4A and PTK2 pair was used for evaluating gene expression in the CL and placenta, whereas all three genes were used for the uterus. The results are presented in Fig. 3. Thus, similar expression patterns were observed for both groups of normalizers. This was also in accordance with previously reported findings [22, 26–28]. Importantly, however, the application of new reference genes resulted in smaller intragroup variation and, therefore, lower *P*-values were observed, e.g., for luteal expression of *IL-1b* and *MHCII*, or uterine expression of *IGF2* and *LHR*.

## Discussion

qPCR is considered to be the most accurate and reliable method for studying gene expression. For proper evaluation of data, reliable reference genes need to be used for normalization of gene expression. Their levels should be stable and not influenced by experimental conditions. This is, however, frequently undervalued and/or overlooked. It becomes even more challenging when morphologically complex organ systems, like the reproductive tract with its continuous hormonal changes, are considered. Moreover, pathological conditions can influence gene expression. Defining potential candidates for reference genes should not be based only on information available from other species. Gene expression profiling by, e.g., microarray or RNA-seq, besides providing biologically important information about differential gene expression, is also a way to provide additional knowledge about highly stable genes. Here, we performed a multistudy analysis of transcriptome data from various experimental setups, including pregnancy and pathological endometrial conditions. Additionally, datasets from antigestagen- and firocoxib-treated dogs were involved. To our knowledge, such an approach has never previously been applied for the dog. Our analysis revealed 1649, 18 and 430 potentially stably-expressed genes for placenta, uterus and CL, respectively. These analyses included all samples, i.e., treated and untreated (placenta and CL), or healthy and diseased (uteri). However, no single gene could be identified for universal use in all tissue types tested. Thus, to decrease intragroup variability in the uterine samples, in the next analysis uterine pathological samples were excluded. Following this, 1994 potentially stable genes were found in all remaining (i.e., healthy) uterine samples and were used in comparison with the remaining tissues. Finally, 36 genes were found to be commonly present in datasets from each tissue type, including CL, placenta and non-pathological uterus. They comprised a group of potential universal references for normalization of gene expression in healthy canine reproductive tissues. From these 36 genes, we chose 4 candidates representing different functional groups, i.e., transcription- and translation-associated factors (*KDM4A*, *EIF4H*), intracellular protein transport (*KDELR2*) or cytoskeleton organization and focal adhesion (*PTK2*). Their stability was tested in 55 available canine samples of placenta, CL and uterus. All selected genes appeared to be more stable than the reference genes used so far. Finally, the three best candidates, i.e., *KDM4A*, *EIF4H*, *PTK2*, were selected. Interestingly, while *KDM4A* and *PTK2* have apparently never been considered for gene expression normalization, the consistent expression of *EIF4H* has been demonstrated in human cancer cells [29], where it was proposed as a reliable control gene. It appears, thus, that *EIF4H* might be a good candidate universal normalizer, however, its application in other species and tissues should be verified.

The usefulness and reliability of the three newly identified candidate genes were further evaluated by applying them to normalize the expression of target genes that were investigated in previous studies [22, 26–28]. The expression of these target genes, which included placental *COX2/PTGS2* and *VEGFA*, luteal *IL-1b* and *MHCII*, and uterine *IGF2* and *LHR*, was normalized and compared with two sets of reference genes: those that were formerly used and our newly proposed genes. Considering that generally less variation was observed within and among the groups, together with lower *P*-values indicating a higher level of significance, we concluded that more accurate findings from expression experiments are possible by applying more stably expressed, newly identified reference genes as internal controls.

Regrettably, as mentioned before, no gene was found to be universal for all tested tissues, including pathological uterine samples. Still, however, our study provides a list of 18 potential candidate genes, which, after verification, could be applied as references for studies performed exclusively on the uterus, including healthy and diseased tissue. Out of these 18 genes we propose several candidates belonging to different functional groups and associated with diverse cellular components that could be subjected to further validation. They include: carboxypeptidase A1 (*CPA1*), an enzyme involved in proteolysis; damage specific DNA binding protein 1 (*DDB1*), a ubiquitous protein participating in a response to DNA damage and repair; and microtubule crosslinking factor 1 (*MTCL1*), a molecule regulating microtubule activity and intracellular transport. Finally, a gene encoding for ribosomal protein L32 (*RPL32*), also called EF-hand calcium binding domain 12 was found among 18 stably expressed genes in the canine uterus. It needs to be mentioned that this molecule, a component of the large ribosomal 60S unit, has been considered before as a reference gene for data normalization. Its high stability has been discussed for application to various tissues in, e.g., pig [30, 31], rat [32], human [33] and chicken [34, 35], and even in several invertebrates [36]. Notably, it was found to be stably expressed in several canine healthy and diseased, i.e., neoplastic, tissues [9, 37, 38], and was therefore proposed as a good reference gene. As for our analysis, its expression was not considered in the present study as it did not meet the inclusion criteria (CV > 0.2) for CL and placenta. Nevertheless, in line with the above cited studies, *RPL32* definitely deserves more attention and should be considered also for uterine tissue.

## Conclusions

In conclusion, with our tissue-targeted analysis, we were able to provide comprehensive lists of potentially stably-expressed genes for target gene expression analyses in reproductive tissues of the female dog. We tested new reference genes, which with exception of *EIF4H*, have never previously been considered for application in canine reproduction studies. With this, new tools have been provided for gene expression quantification studies. Interestingly, when including pathological uterine samples, no unique gene was found for all tissues and conditions evaluated herein. However, several reference genes that are highly reliable for normalization of data from canine CL, placenta and healthy uterus were found. Further, when analyzing only uterine samples, we have identified 18 potentially stable genes, which could become useful for studies involving pathological conditions such as pyometra. Of these, *RPL32* in particular deserves more attention in future, as its stability was previously confirmed in multiple tissues and species, including the dog. Apart from *RPL32*, we propose several other candidate genes that should be validated in the future and considered for diseased uterine tissues (e.g., *CPA1*, *DDB1 MTCL1).*

Based on our results, we propose to use *KDM4A*, *PTK2* for normalization of data obtained from canine CL and placenta. As for the healthy uterus, the inclusion of a third reference gene, *EIF4H*, is suggested. By applying the proposed approach, more reliable interpretation of qPCR data can be achieved, leading to better understanding of canine reproductive physiology as well as of mechanisms leading to uterine pathological conditions.

## Methods

### Data collection

The following raw data from microarray and RNA-seq experiments performed on canine reproductive tissues (ovary, CL, uterus, placenta) were included in our multistudy analysis (for datasets already described in the available literature and deposited in NCBI’s Gene Expression Omnibus, GEO Series accession numbers are given; the total number of samples used in each study is given in parentheses). Our in-house datasets (1) GSE126031 – the dataset derived from analysis of the canine placental transcriptome by Nowak et al. [39], which includes samples of canine placenta from the mid-gestation stage, prepartum luteolysis and antigestagen (aglepristone) - induced luteolysis (*n* = 9); (2) GSE98657 - the RNA-seq study by Zatta et al. [27] that included corpora lutea (CL) from clinically healthy bitches, which were assigned to the following experimental groups: mid-pregnancy (days 35–40), active prepartum luteolysis, antigestagen-treated mid-gestation group (days 40–45) and non-pregnant bitches at late luteal regression (day 65 after ovulation), (*n* = 15); (3) the microarray study by Graubner et al. [40] which included uterine samples of non-pregnant and early pregnant dogs at the pre-implantation stage (days 10–12) of pregnancy; this dataset [40] has not been deposited in GEO, however, it is available as publication supplemental files and included *n* = 14 of samples; (4) GSE130369 - the dataset from our most recent study, Tavares Pereira et al. [41], which contains RNA-seq transcriptome data from luteal samples of dogs treated with a non-steroidal anti-inflammatory drug (NSAID), cyclooxygenase-2 (COX2/PTGS2) inhibitor, firocoxib, at days 5, 10, 20, 30 after ovulation, and their non-treated controls (*n* = 24). Additionally, we have included the following data available from the literature: (5) GSE69481 - the microarray study by Voorwald et al. [42] which included fresh endometrium samples collected from 21 healthy female dogs during diestrus, 16 with cystic endometrial hyperplasia (CEH), 15 with mucometra and 17 with pyometra (eight open and nine closed-cervix) (*n* = 69); (6) GSE17878 - the microarray study by Hagman et al. [43] which included uteri from dogs with pyometra compared to healthy dogs (*n* = 8); (7) GSE99877 - the microarray study by Bukowska et al. [44] included uteri from clinically healthy bitches and bitches with pyometra (20 clinically healthy, 23 with pyometra). RNA used for that study was pooled into four separate vials for control and pyometra (n = 8).

Thus, in summary, 153 samples were analyzed, including 45, 99 and 9 samples from the CL, uterus and placenta, respectively. Of the 99 uterine samples, 56 represented pathological conditions, i.e., pyometra, hydrometra or CEH.

### Determination of potential candidate reference genes

The collected data were subjected to analysis and the following inclusion criteria were considered for each detected gene in a given dataset: (1) the mean expression level of each gene, i.e., the base mean value of number of transcripts, was set to > 500. This step removed underrepresented genes and assured minimal detection levels for qPCR reactions; (2) the coefficient of variation (CV), i.e., the ratio of standard deviation (SD) and mean, which gives information about the extent of variability in gene expression datasets. For this study, a threshold CV of < 0.2 was applied. Following filtering of the genes that fulfilled the above-listed criteria, comparative analysis was performed. First, lists of potential stably-expressed genes were generated for each tissue type. Subsequently, these lists were compared with each other in order to determine universal reference genes in all examined tissues and conditions.

### Evaluation of stability of candidate reference genes

It needs to be emphasized that all computing analyses, as presented above, were done in order to narrow down the extensive gene lists and to define potential candidates for reliable reference genes. They could not, however, be treated as being definitive without verifying their stability by qPCR in specific tissues. For this reason, TaqMan RT-qPCR assays were applied to validate the expression of genes in available canine reproductive tissues from our previous studies [27, 28, 40, 45]. Therefore, 55 samples of different organ origin, i.e., with high diversity, were used. Of these, 23 were derived from CL, 19 from uterus and 13 from placenta. The full list of samples used for evaluation experiments, including details regarding their origin and ethical approvals, is provided in Supplementary Material 1. These sample-sets contained tissues from non-pregnant and pregnant animals, including different stages of gestation, and from animals subjected to different treatments (aglepristone-induced luteolysis, firocoxib treatment). Next, the expression and stability of genes were evaluated. These experiments included selected potential candidates and three reference genes used routinely until now in previous studies, i.e., *GAPDH*, *β-actin* and *cyclophillin A* (*PPIA*). The stability expression values of all genes were calculated, and subsequently genes were ranked using online tools: RefFinder [46] and NormFinder softwares [47]. The RefFinder algorithm integrates 4 commonly-used stability evaluation programs: the comparative delta-Ct method [48], BestKeeper [49], NormFinder [47] and GeNorm [25], to generate a comprehensive ranking by calculating the geometric mean. Additionally, NormFinder [47] was used to perform estimations of the intra- and inter-group expression variations for each subgroup of samples to provide the most reliable combination of candidate reference gene pairs. Finally, a Microsoft Excel add-in, GeNorm [25] was applied to compute pairwise variation V_n/n + 1_, which is a parameter that informs how many reference genes should be used for normalization. Briefly, V_n/n + 1_ gives information about whether addition of another gene would have a significant effect on data normalization, and a high variation implies that an additional gene will have a significant effect and preferably should be included. As advised by Vandesompele and coworkers [25], using 0.15 as a cut-off is considered to be reliable. Thus, the values below this cut-off indicate a lack of need to include an additional gene.

### Validation of proposed reference genes by TaqMan RT-qPCR

To verify the suitability of newly-identified reference genes, we investigated the expression of target genes whose expression is known to vary in selected reproductive tract tissues of female dogs. The expression of target genes was normalized using either the conventional set of reference genes (*GAPDH*, *β-actin* and *cyclophillin A*/*PPIA*) or newly-found candidates. Thus, following information available in the literature, placental expression of cyclooxygenase-2 (*COX2/PTGS2*) [22] and vascular endothelial growth factor A (*VEGFA*) [26] was re-evaluated in groups comprising mid-gestation (*n* = 5), prepartum luteolysis (*n* = 3) and antigestagen-induced luteolysis (n = 5; see Supplementary Material 1 for details). For the CL, we examined the expression of interleukin 1b (*IL-1b*) [27] and major histocompatibility complex class II molecules (*MHCII*) [27] between mid-gestation (n = 5) and prepartum luteolysis (n = 3) groups. Finally, the expression of insulin-like growth factor 2 (*IGF2*) [28] and luteinizing hormone receptor (*LHR*) [28] was evaluated in the non-pregnant uterus (Embryo minus, n = 5) and at the pre-implantation stage (Embryo plus, n = 5).

### Total RNA extraction, reverse transcription, semi-quantitative real time TaqMan RT-qPCR and data evaluation

Total RNA from frozen (− 80 °C) tissues was isolated, using TRIzol reagent based on the manufacturer’s protocol (Invitrogen, Carlsbad, CA, USA) and as previously described [50, 51]. The quality and quantity of extracted RNA was verified with a NanoDrop 2000C spectrophotometer (Thermo Fisher Scientific AG, Reinach, Switzerland). Isolated RNA was subjected to DNase treatment in order to remove any genomic DNA contamination. The RQ1 Rnase-free Dnase from Promega (Duebendorf, Switzerland) was used following the manufacturer’s protocol. Reverse transcription (RT) was performed as described before [50, 51], using random hexamers as primers. All RT reagents were purchased from Applied Biosystems by Thermo Fisher (Carlsbad, CA, USA). The RT reactions were done in an Eppendorf Mastercycler (Vaudaux-Eppendorf AG, Basel, Switzerland). Canine-specific primers and probe mixtures for proposed candidate genes, i.e., *KDM4A*, *KDELR2*, *EIF4H* and *PTK2*, as well as for two previously used reference genes, *β-actin* and *cyclophillin A/PPIA*, and *IGF2*, were commercially available and purchased from Applied Biosystems. For other evaluated genes, i.e., *GAPDH*, *COX2/PTGES*, *IL-1b*, *LHR*, *MHCII*, *VEGFA*, primers and TaqMan probes labeled with 6-carboxyfluorescein (6-FAM) and 6-carboxytetramethylrhodamine (TAMRA), were designed with Primer Express Software ver. 2.0 (Applied Biosystems) and purchased from Microsynth (Balgach, Switzerland). A complete list of predesigned assays and sequences of primers and TaqMan probes is provided in Table 1. The efficiencies of the PCR assays were determined by the CT slope method assuring approximately 100% reaction efficiency.

**Table 1:** List of predesigned assays and in-house designed primers and probes used for real time TaqMan qPCR

**Gene**	**Gene name**	**Accession number**	**Sequence**	**Amplicon length**
*COX2/PTGS2*	cyclooxygenase-2	HQ_110882	Forward: 5′-GGA GCA TAA CAG AGT GTG TGA TGT G-3’	87 bp
			Reverse: 5′-AAG TAT TAG CCT GCT CGT CTG GAA T-3’	
			Probe: 5′-CGC TCA TCA TCC CAT TCT GGG TGC T-3’	
*GAPDH*	glyceraldehyde-3-phosphate dehydrogenase	AB_028142	Forward: 5′-GCT GCC AAA TAT GAC GAC ATC A-3’	75 bp
			Reverse: 5′-GTA GCC CAG GAT GCC TTT GAG-3′	
			Probe: 5′-TCC CTC CGA TGC CTG CTT CAC TAC CTT-3’	
*IL-1b*	interleukin 1b	NM_001037971	Forward: 5′-TGC CAA GAC CTG AAC CAC AGT-3′	97 bp
			Reverse: 5′-CTG ACA CGA AAT GCC TCA GAC T-3′	
			Probe: 5′-CAT CCA GTT GCA AGT CTC CCA CCA GC-3′	
*LHR*	luteinizing hormone receptor	XM_538486	Forward: 5′-TCA TCA TTT GTG CTT GCT ACA TTA AA-3’	98 bp
			Reverse: 5′-CGC CAT TTT CTT AGC AAT CTT TG-3’	
			Probe: 5′-TGC AGT TCA AAA TCC AGA GCT GAT GGC-3’	
*MHCII*	major histocompatibility complex class II	NM_001011723	Forward: 5′-GGA GAG CCC AAC ATC CTC ATC-3′	90 bp
			Reverse: 5′-GGT GAC AGG GTT TCC ATT TCG-3′	
			Probe: 5′-TCG ACA AGT TCT CCC CAC C-3′	
*VEGFA*	vascular endothelial growth factor A	NM_001003175	Forward: 5′-GTG CCC ACT GAG GAG TTC AAC-3’	72 bp
			Reverse: 5′-CCC TAT GTG CTG GCC TTG AT-3’	
			Probe: 5′-CAC CAT GCA GAT TAT GCG GAT CAA ACC-3’	
**Gene**		**Gene name**		**Product no.**
*β-actin*		actin beta		Cf03023880_g1
*cyclophilin A/PPIA*		cyclophilin A		Cf03986523_gH
*EIF4H*		eukaryotic translation initiation factor 4H		Cf02713640_m1
*IGF2*		insulin-like growth factor 2		Cf02647136_m1
*KDELR2*		endoplasmic reticulum lumen protein-retaining receptor 2		Cf02668050_m1
*KDM4A*		lysine-specific demethylase 4A		Cf02708629_m1
*PTK2*		focal adhesion kinase 1, protein tyrosine kinase 2		Cf02684608_m1

For all TaqMan RT-qPCR experiments, an automated fluorometer ABI Prism 7500 Sequence Detection System (Applied Biosystems) was used and the following amplification conditions were applied: initial denaturation for 10 min at 95 °C, followed by 40 cycles each for 15 s at 95 °C and 1 min at 60 °C. The 25 μl reaction mixture included: 200 nM TaqMan Probe, 300 nM of each primer, 12.5 μl Fast Start Universal Probe Master (ROX) (Roche Diagnostics) and 5 μl of cDNA corresponding to 100 ng total RNA. Each sample was run in duplicates. Autoclaved water and the so-called minus-RT controls (i.e., samples treated with DNase but not subjected to RT) instead of cDNA were used as negative controls. The expression of target genes, i.e., *COX2/PTGS2*, *VEGFA*, *MHCII*, *IL-1b*, *IGF2* and *LHR* was normalized according to either the three new proposed genes, i.e., *KDM4A*, *EIF4H*, *PTK2* or three previously used reference genes: *GAPDH*, *β-actin* and *cyclophillin A/PPIA* and calculated using the comparative CT method (ΔΔCT method) as reported previously [50, 51].

### Statistics

Statistical analysis was performed using the software program GraphPad 3.06 (GraphPad Software). An unpaired, two-tailed Student’s t-test was performed to compare the levels of luteal *IL-1b* and *MHCII* between mid-gestation and prepartum luteolysis groups, and uterine *IGF2* and *LHR* in non-pregnant and pre-implantation uteri. Parametric one-way analysis of variance (ANOVA) was performed followed by a Tukey–Kramer multiple comparisons post-test to compare the levels of placental *COX2/PTGS2* and *VEGFA* mRNA expression between mid-gestation, antigestagen-induced abortion/luteolysis and prepartum luteolysis groups. *P* < 0.05 was considered statistically significant.

## Supplementary Information

**Additional file 1: Supplementary Material 1.** List of samples used to validate stability of candidate reference genes.

**Additional file 2: Supplementary Material 2.** Lists of stably expressed genes determined for canine reproductive tissues (placenta, corpus luteum, uterus).

## Data Availability

The datasets used for analysis during the current study are available under: (1) Nowak et al. [39]: GSE126031: https://www.ncbi.nlm.nih.gov/geo/query/acc.cgi?acc=GSE126031 (2) Zatta et al. [27] GSE98657: https://www.ncbi.nlm.nih.gov/geo/query/acc.cgi?acc=GSE98657 (3) Graubner et al. [40] https://www.ncbi.nlm.nih.gov/pmc/articles/PMC5803782/ (4) Tavares Pereira et al. [41] GSE130369: https://www.ncbi.nlm.nih.gov/geo/query/acc.cgi?acc=GSE130369 (5) Voorwald et al. [42] GSE69481: https://www.ncbi.nlm.nih.gov/geo/query/acc.cgi?acc=GSE69481 (6) Hagman et al. [43] GSE17878: https://www.ncbi.nlm.nih.gov/geo/query/acc.cgi?acc=GSE17878 (7) Bukowska et al. [44] GSE99877: https://www.ncbi.nlm.nih.gov/geo/query/acc.cgi?acc=GSE99877
